# Response: Safety and adverse events following COVID‐19 vaccination among people with epilepsy: Correspondence

**DOI:** 10.1002/epi4.12675

**Published:** 2022-12-11

**Authors:** Marjorie Jia Yi Ong, Ching Soong Khoo, Yi Xuan Lee, Vaanee Poongkuntran, Chia Khoi Tang, Yu Joe Choong, Rozita Hod, Hui Jan Tan

**Affiliations:** ^1^ Neurology Unit, Department of Medicine Universiti Kebangsaan Malaysia Medical Centre Kuala Lumpur Malaysia; ^2^ Department of Community Health Universiti Kebangsaan Malaysia Medical Centre Kuala Lumpur Malaysia

**Keywords:** adverse events following immunisation, COVID‐19 vaccinations, Epilepsy

Dear Dr. Rujittika Mungmunpuntipantip,

Thank you for your letter and comments on the paper. The paper described by Ong et al. focuses on the safety and adverse events among the PWE following COVID‐19 immunization. Some of the recruited subjects in the paper have other comorbidities, which do not deter them from being vaccinated. Some adverse events are inevitable, which are not proven to be more frequent or more severe compared with the general population. All in all, the benefits of COVID‐19 vaccination outweigh its potential risks.

We confirm that we have read the Journal's position on issues involved in ethical publication and affirm that this report is consistent with those guidelines. Neither of the authors has any conflict of interest to disclose for this letter.

Thank you.

Best regards,

Corresponding author:

Ching Soong Khoo.

Department of Medicine,

Universiti Kebangsaan Malaysia Medical Centre.

Email: chingsoongkhoo@gmail.com

Lead author:

Marjorie Jia Yi Ong.

The National University of Malaysia.

Email: marjorieongjiayi@gmail.com

